# Predictors of Seatbelt Use Among Saudi Adults: Results From the National Biobank Project

**DOI:** 10.3389/fpubh.2020.579071

**Published:** 2020-10-26

**Authors:** Suliman Alghnam, Mesnad Alyabsi, Alhanouf Aburas, Taif Alqahtani, Miasem Bajowaiber, Ali Alghamdi, Ada Alqunaibet

**Affiliations:** ^1^Population Health Section, King Abdullah International Medical Research Centre (KAIMRC), Riyadh, Saudi Arabia; ^2^King Saud Bin Abdulaziz University for Health Sciences (KSAU-HS), Riyadh, Saudi Arabia; ^3^Public Health Department, Ministry of Health, Riyadh, Saudi Arabia; ^4^National Centre for Traffic Safety, Ministry of Transportation, Riyadh, Saudi Arabia; ^5^Saudi Centre for Disease Control and Prevention, Riyadh, Saudi Arabia

**Keywords:** seatbelt, seat belt laws, driver behavior, unrestrained, disability, fatality, Saudi, road traffic accidents

## Abstract

**Introduction:** Road traffic crashes (RTCs) are a leading cause of death and disabilities and impose a significant burden on the healthcare system and economy of Saudi Arabia. Around 20% of all hospital beds are occupied by victims of RTCs, which represent approximately 80% of trauma deaths occurring in these facilities. Using a seatbelt is an effective method to reduce traffic deaths and minimize the extent of associated injuries. However, little is currently known about the prevalence and predictors of seatbelt use in Saudi Arabia. More studies are needed to determine the trends of seatbelt use and study the relationship between individual factors and compliance with seatbelt use laws. The aim of the present study is to examine the prevalence and predictors of seatbelt use using the National Saudi Biobank dataset.

**Materials and Methods:** This cross-sectional study was conducted using an in-person survey from the Saudi National Biobank (SNB). The participants were adults affiliated with the Ministry of National Guard Health Affairs in Riyadh who were examined between 2017 and 2019. Chi-squared and Wald tests were used to assess the association between the respondents' characteristics and their seatbelt use. In addition, logistic regression models were constructed to assess the univariate and multivariate associations between seatbelt use and potential predictors. All statistical tests were two-sided, and the findings were considered significant at *P* < 0.05.

**Results:** A total of 5,790 adults participated in the survey. The majority of the participants (52.44%) were between 18 and 25 years old, half were males, and 58.80% were single. About 42.83% of the participants reported consistent seatbelt use as drivers or passengers. In the multivariable analysis, females were 86% less likely to wear seatbelts than males (OR = 0.136, 95%CI = 0.107–0.173). Individuals who rated their mental health as “weak” were 26% less likely to wear seatbelts than those who reported “excellent” mental health status.

**Conclusion:** Seatbelt use remains low in the country and substantially lower than in developed countries. Young adults, females, and individuals reporting suboptimal mental health were less likely to fasten their seatbelts. These findings are valuable for public health programs to target specific groups and raise awareness about the need to increase seatbelt compliance and reduce traffic injuries.

## Introduction

Road traffic crashes (RTCs) are a leading cause of death, disabilities, and healthcare loss worldwide (1). The global cost of RTCs between 2015 and 2030 is projected to reach $1.8 trillion, amounting to 0.12% of the global gross domestic product (GDP) (2). In the Kingdom of Saudi Arabia (KSA), around 20% of all hospital beds are occupied by victims of RTCs, who represent approximately 80% of trauma deaths (3). The local burden of RTCs in developing countries is even greater (1). A study comparing RTCs in the United States (US) and KSA found that while only 2% of RTCs in the US are fatal, 23% are fatal in KSA (4). However, more recent estimates suggest that only 15% of RTCs in Saudi Arabia are fatal (5). Nonetheless, estimates indicate that they result in losses in the workforce, hospital resources, and human capital. All of these constitute a significant burden on the Saudi population, the economy, and public health (6).

Various factors influence the risk of fatal crashes, including weather, road conditions, an absence of emergency care services, and reckless driving (7). Excessive speeding is a leading risk factor for RTCs in the KSA (8), and cellphones are commonly used while driving in Riyadh, Saudi Arabia, which presents a significant risk for crashes and results in disability (9). Moreover, failing to use seatbelts is known as a risk factor for fatal injuries resulting from RTCs in KSA (10). Factors that facilitate traffic safety include improving the design or conditions of roads, strictly implementing traffic laws, enhancing the response to crashes, and improving the conditions of vehicles (11).

Responsible driving is among the most effective and efficient preventive behaviors against RTCs and includes adhering to the speed limit, following traffic signals, cellphone-free driving, and correctly using seatbelts. Thus, KSA has installed surveillance cameras to detect a lack of adherence in an effort to promote safe driving behaviors, which has been associated with an increase in compliance with traffic laws in Riyadh (12). The rate of seatbelt use while driving is low in KSA, with only 60% of drivers and 22.7% of front-seat passengers using them in 2005 (13). This rate has deteriorated in the following 2 years, with the rate of seatbelt use decreasing to 27.8% among drivers and 14.7% among front-seat passengers (14). More recent reports indicate that 10.3% of Saudi drivers use seatbelts (15).

Alghnam et al. (10) found that only a third of drivers in Riyadh (34%) use seatbelts. Low rates of seatbelt use were also reported in the Eastern Province of KSA, ranging from 43 to 47% for drivers and 26–30% for front-seat passengers, with even lower rates for rear-seat users (16). However, most of the studies done to estimate the prevalence of seatbelt use in KSA are out-dated, and there are substantial differences in the reported estimates (12). Therefore, a more updated and comprehensive analysis is warranted to better inform policymakers, researchers, and the public.

Various factors may influence the likelihood of using seatbelts in KSA. In line with international literature, seatbelt use is likely to be associated with socioeconomic factors. Alghnam et al. (10) found that drivers in affluent neighborhoods are three times more likely to wear seatbelts than those in less affluent areas. Increasing police inspection and using surveillance cameras are other predictors of seatbelt use, which are likely altering drivers' behaviors in KSA and other countries (17). Education is another factor that could affect the rate of compliance. People who are more educated about the importance of seatbelt use are more likely to utilize them (18).

Currently, little is known about the current prevalence and predictors of compliance with seatbelt use laws in KSA. More studies are needed to reflect the trends of seatbelt use and to study the relationship between individual factors and the Saudi population's compliance with seatbelt use laws. Thus, the aim of this study is to estimate the prevalence and predictors of seatbelt use among Saudi individuals.

## Methods

This cross-sectional study was performed using data from the Saudi National Biobank (SNB). The SNB is an on-going project with the aim of understanding the current health behaviors of the Saudi population. This is accomplished using a combination of bio-specimens (blood, DNA, tissue, and biopsy) and survey data, such as sociodemographic and medical history information. The project was designed to investigate the epidemiology of diseases comprehensively in the KSA.

The study utilized data collected via the surveys as part of the SNB. The in-person survey included individuals served by the Ministry of National Guard Health Affairs (MNG-HA) in Riyadh between the years 2017 and 2019. This population included military service personnel and their families, members of the MNG-HA population (patients or healthy volunteers), health care workers, and students from the MNG-HA-related healthcare system. The MNG-HA population (>1 million individuals) is served by tertiary-care hospitals and four main primary- and secondary-care clinics. Additionally, MNG-HA electronic health data of consenting participants were used to complement the participants' information. Participants provided consent for the SNB data collection and access to their healthcare records to link participants' characteristics with their long- and short-term healthcare outcomes.

### Survey Development and Administration

The SNB research team created the survey content based on previously developed and validated questionnaires. The SNB research team subjected the preliminary survey questions to pilot testing, and the items were revised according to the findings. The survey consists of the following sections: the date and location of recruitment, demographic information, general health status, personal and family medical history, health behaviors, women and men's health, and anthropometric measurements.

Survey items are primarily closed-ended questions with Likert-scale responses. A 5-point Likert-scale rating was used to assess overall health status with the designations “excellent,” “very good,” “good,” “fair,” and “weak.” Likewise, the time spent on activities such as walking was evaluated using a 5-point Likert-scale rating with the designations “never,” “a few times,” “sometimes,” “most times,” and “all the time.” Surveys were administered to participants by trained research coordinators. Before the participants provided consent, the coordinators described the Biobank's objectives to them, the benefits of participation, the security and privacy of the collected information, and the participants' rights.

### Study Population and Data Extraction

Since the study is focused on participants' seatbelt practices, individuals included in the study were those who responded to the survey between November 1, 2017, and January 1, 2019. Participants were aged 18 years or older. The outcome was based on the question, “do you use a seatbelt while driving or riding in a car?” The response options to the seatbelt question were “always,” “sometimes,” “rarely,” “never,” “refuse to answer,” and “do not know.” We dichotomized the outcome variable into “yes” if the response was “always” and “no” otherwise since the purpose of the study is to investigate habitual use.

Sociodemographic data were also extracted, including age, marital status, education level, occupation, and household income. Other factors were included, such as smoking status, overall physical and mental health status, disabilities, dentist visits, physical activities, and comorbidities. The men's level of vigorous, moderate, or light exercise was measured using values between 1, which indicates never having exercised, and 9, which means they had done ≥8 exercise sessions during the past week. The exercise variable was categorized into “never,” “once,” “twice,” or “more than twice.” The study was reviewed and approved by the Institutional Review Board of King Abdullah International Medical Research Centre.

### Statistical Analysis

Chi-squared tests were used to assess demographics, general health status, personal and family medical history, health behaviors, and anthropometric measurement characteristics. Wald tests were used to determine the association between the mentioned covariates and seatbelt use. Logistic regression models were used to determine the bivariate and multivariate associations between seatbelt use and covariates. Backward elimination was used in the multivariate analysis to retain all variables with *P* ≤ 0.20. All statistical tests were two-sided, and the findings were considered statistically significant at *P* < 0.05. All analyses were conducted using the statistical software SAS version 9.4 (SAS Institute Inc., Cary, NC).

## Results

A total of 5,790 individuals participated in the SNB between 2017 and 2019. Gender was equally split, and most participants were single (58.80%) and between the ages of 18–25 years (52.44%). Of the total study population, 2,480 respondents (42.83%) reported regular seatbelt use when driving or riding in a car. Among those who reported wearing seatbelts, over two-thirds were males, 57.16% were single, half were employed, and half had a college education (Table 1). Around 2.7% (*n* = 158) of the study population reported a cancer diagnosis, of which 68 individuals reported habitual seatbelt use. At least four out of five participants reported very good or excellent overall, physical, and mental health during the past 2 weeks. About 18% of the population reported that they are current smokers. Of the participants who reported weak mental health status (*n* = 44), 29 individuals reported not using seatbelts, while the remaining reported habitual seatbelt use.

**Table 1:** Characteristics of the Biobank population stratified by seatbelt use.

**Characteristics**	**Total (5,790)**	**Seatbelt (2,480)Missing (43)**	**No seatbelt (3,310)**
**Age**			
18–25	3,036 (52.44)	1,253 (50.52)	1,783 (53.87)
26–45	2,454 (42.38)	1,084 (43.71)	1,370 (41.39)
≥46	300 (5.18)	143 (5.77)	157 (4.74)
Median (IQR)	25 (12.0)	25 (13.0)	25 (12.0)
**Gender**			
Male	2,870 (49.20)	1,704 (68.01)	1,146 (34.62)
Female	2,963 (50.80)	776 (31.29)	2,164 (65.38)
**Marital status (missing** **=** **4)**			
Never married	3,402 (58.80)	1,417 (57.16)	1,985 (60.02)
Married	2,255 (38.97)	1,026 (41.39)	1,229 (37.16)
Divorced, separated or widowed	129 (2.23)	36 (1.45)	93 (2.81)
**Employment status (missing** **=** **22)**			
Employed	2,634 (45.16)	1,223 (49.55)	1,391 (42.14)
Unemployed	266 (4.56)	94 (3.81)	171 (5.18)
Student	2261 (38.76)	957 (38.78)	1288 (39.02)
Parenting	549 (9.41)	139 (5.63)	406 (12.30)
Retired/others	100 (1.73)	55 (2.23)	45 (1.36)
**Family income (SAR) (missing** **=** **727)**			
5,000	960 (18.93)	351 (16.01)	609 (21.15)
5,000–10,000	1,219 (24.03)	481 (21.93)	738 (25.63)
10,001–15,000	906 (17.86)	416 (18.97)	490 (17.02)
15,001–20,000	642 (12.66)	288 (13.13)	354 (12.30)
>20,000	1,345 (26.52)	657 (29.96)	688 (23.90)
**Residence (missing** **=** **16)**			
Own	3,533 (61.19)	1,525 (61.64)	2,008 (60.85)
Rental	2,096 (36.30)	897 (36.26)	1,199 (36.33)
Other arrangements	145 (2.51)	52 (2.10)	93 (2.82)
**Education level (missing** **=** **19)**			
Primary school	173 (3.00)	51 (2.06)	122 (3.70)
Intermediate or high school	, (48.55)	1,166 (47.21)	1,636 (49.56)
College or higher education	2,796 (48.45)	1,253 (50.73)	1,543 (46.74)
**Overall health (missing** **=** **39)**			
Excellent	3,137 (54.53)	1,450 (58.73)	1,687 (51.37)
Very good	1,729 (30.05)	700 (28.35)	1,029 (31.33)
Good/Okay	848 (14.74)	303 (12.27)	545 (16.60)
Weak	39 (0.68)	16 (0.65)	23 (0.70)
**Mental health (missing** **=** **15)**			
Excellent	1,845 (31.94)	899 (36.34)	946 (28.65)
Very good	2,775 (48.04)	1,145 (46.28)	1,630 (49.36)
Good	1,008 (17.45)	374 (15.12)	634 (19.20)
Okay	104 (1.80)	41 (1.66)	63 (1.91)
Weak	44 (0.75)	15 (0.61)	29 (0.88)
**Physical health (missing** **=** **17)**			
Excellent	2,038 (35.30)	915 (36.98)	1,123 (34.03)
Very good	2,876 (49.81)	1,230 (49.72)	1,646 (49.88)
Good	792 (13.66)	309 (12.49)	483 (14.64)
Okay	48 (0.83)	11 (0.44)	37 (1.12)
Weak	20 (0.35)	9 (0.36)	11 (0.33)
**Body mass index (missing)**			
Underweight	208 (7.43)	81 (6.72)	127 (7.97)
Normal	1,120 (40.0)	472 (39.14)	648 (40.65)
Overweight	787 (28.11)	331 (27.45)	456 (28.61)
Obese	685 (24.46)	322 (26.70)	363 (22.77)
**Depression (missing** **=** **92)**			
Never	2,289 (40.16)	1,122 (46.04)	1,167 (35.76)
Some days	2,231 (39.14)	851 (34.92)	1,380 (42.29)
Few days	713 (12.51)	275 (11.28)	438 (13.42)
More than few days	467 (8.19)	189 (7.76)	278 (8.52)
**Anxiety (missing** **=** **82)**			
Never	1,896 (33.20)	917 (37.51)	979 (29.98)
Some days	2,201 (38.55)	907 (37.10)	1,294 (39.63)
Few days	910 (15.94)	354 (14.48)	556 (17.03)
More than few days	703 (12.31)	267 (10.92)	436 (13.35)
**Disability (missing** **=** **56)**			
Yes	116 (2.02)	54 (2.20)	62 (1.89)
No	5,618 (97.98)	2,401 (97.80)	3,217 (98.11)
**Brushing (missing** **=** **89)**			
Never	372 (6.51)	171 (6.98)	201 (6.16)
Once a day	1,768 (30.94)	830 (33.86)	938 (28.74)
Twice a day	2,631 (46.04)	1,060 (43.25)	1,571 (48.13)
More than twice a day	944 (16.52)	390 (15.91)	554 (16.97)
**Dentist visits (missing** **=** **67)**			
Yes	3,608 (62.89)	1,548 (62.95)	2,060 (62.84)
No	2,129 (37.11)	911 (37.05)	1,218 (37.16)
**Vigorous exercises (missing** **=** **15)**			
Never	2,144 (37.11)	850 (34.34)	1,294 (39.19)
Once a day	2,465 (42.67)	1,082 (43.72)	1,383 (41.88)
Twice a day	704 (12.19)	324 (13.09)	380 (11.51)
More than twice a day	464 (8.03)	219 (8.85)	245 (7.42)
**Moderate exercises (missing** **=** **19)**			
Never	4,223 (73.14)	1,715 (69.29)	2,508 (76.02)
Once a day	495 (8.57)	259 (10.46)	236 (7.15)
Twice a day	284 (4.92)	137 (5.54)	147 (4.46)
More than twice a day	772 (13.37)	364 (14.71)	408 (12.37)
**Light exercises (missing** **=** **19)**			
Never	3,663 (63.44)	1,494 (60.41)	2,169 (65.71)
Once a day	530 (9.18)	238 (9.62)	292 (8.85)
Twice a day	443 (7.67)	216 (8.73)	227 (6.88)
More than twice a day	1,138 (19.71)	525 (21.23)	613 (18.57)
**Smoking (missing** **=** **149)**			
Yes	1,018 (18.03)	504 (20.94)	514 (15.86)
No	4,629 (81.97)	1,903 (79.06)	2,726 (84.14)
**Shisha use (missing** **=** **27)**			
Yes	479 (8.31)	236 (9.55)	243 (7.38)
No	5,286 (91.69)	2,236 (90.45)	3,050 (92.62)
**Cigar use (missing** **=** **23)**			
Yes	51 (0.88)	22 (0.89)	29 (0.88)
No	5,717 (99.12)	2,452 (99.11)	3,265 (99.12)
**E-Cigarettes use (missing** **=** **32)**			
Yes	55 (0.95)	35 (1.42)	20 (0.61)
No	5,705 (99.05)	2,436 (98.58)	3,269 (99.39)
**Chewing tobacco (missing** **=** **36)**			
Yes	18 (0.31)	4 (0.16)	14 (0.43)
No	5,739 (99.69)	2,467 (99.84)	3,272 (99.57)
**Chronic illnesses**			
Diabetes mellitus	341 (5.90)	165 (6.66)	176 (5.33)
hypertension	246 (4.26)	125 (5.05)	121 (3.67)
Hypercholesterolemia	432 (7.48)	194 (7.83)	238 (7.21)
CVD	120 (2.08)	62 (2.50)	58 (1.76)
Arthritis	260 (4.50)	89 (3.59)	171 (5.18)
Hepatitis	91 (1.57)	49 (1.98)	90 (2.72)
Cancer	158 (2.73)	68 (2.74)	90 (2.72)
Depression	110 (1.91)	41 (1.66)	69 (2.09)
Anxiety	81 (1.40)	31 (1.25)	50 (1.51)
Asthma	281 (4.86)	124 (5.01)	157 (4.76)

Table 2 shows the results of the bivariate analysis of the association between wearing seatbelts and participants' characteristics. The following variables were associated with seatbelt use at the univariable level: age, gender, marital status, employment status, family income, education level, overall mental and physical health, body mass index (BMI), depression or anxiety in the past 2 weeks, vigorous, moderate, or light exercise, and the use of tobacco, shisha, or e-cigarettes.

**Table 2:** The univariate and multivariable association between sociodemographic characteristics and seatbelt use among Saudi participants.

	**Seatbelt use**				
**Sociodemographic factors**	**Univariate**			**Multivariate**	
	**OR**	**95% CI**	***P-*value**	**AOR**	**95% CI**
**Age**					
18–25	1.0		0.02	1.0	
26–45	**1.126**	**(1.011, 1.254)**		**1.490**	**(1.103, 2.012)**
≥46	**1.296**	**(1.022, 1.644)**		1.444	(0.814, 2.560)
**Gender**					
Male	1.0		<0.001	1.0	
Female	**0.241**	**(0.216, 0.269)**		**0.136**	**(0.107, 0.173)**
**Marital status**					
Never married	1.0		<0.001	1.0	
Married	**1.169**	**(1.050, 1.302)**		**1.3833**	**(1.044, 1.832)**
Divorced, separated or widowed	**0.543**	**(0.367, 0.802)**		1.258	(0.654, 2.420)
**Employment status**					
Employed	1.0		<0.001	1.0	
Unemployed	**0.625**	**(0.481, 0.813)**		**1.593**	**(1.031, 2.461)**
Student	**0.845**	**(0.754, 0.947)**		**1.829**	**(1.351, 2.478)**
Parenting	**0.389**	**(0.316, 0.479)**		0.940	(0.617, 1.432)
Retired	1.390	(0.931, 2.077)		1.005	(0.463, 2.184)
**Family income**					
5,000	1.0		<0.001	1.0	
5,000–10,000	1.131	(0.950, 1.346)		0.947	(0.693, 1.294)
10,001–15,000	**1.473**	**(1.224, 1.773)**		0.981	(0.706, 1.363)
15,001–20,000	**1.412**	**(1.152, 1.730)**		1.335	(0.959, 1.858)
>20,000	**1.657**	**(1.399, 1.963)**		1.309	(0.985, 1.740)
**Residence**					
Own	1.0		0.22		
Rental	0.985	(0.883, 1.099)			
Other arrangements	0.736	(0.521, 1.040)			
**Education level**					
Primary school	1.0		0.0001	1.0	
Intermediate or high school	**1.704**	**(1.219, 2.383)**		1.399	(0.688, 2.841)
College or higher education	**1.942**	**(1.389, 2.715)**		1.946	(0.948, 3.995)
**Overall health**					
Excellent	1.0		<0.001		
Very good	**0.791**	**(0.703, 0.891)**			
Good/okay	**0.647**	**(0.553, 0.757)**			
Weak	0.809	(0.426, 1.538)			
**Mental health**					
Excellent	1.0		<0.001	1.0	
Very good	0.544	(0.290, 1.022)		0.737	(0.238, 2.284)
Good	0.685	(0.457, 1.025)		0.798	(0.403, 1.578)
Okay	**0.621**	**(0.531, 0.726)**		**0.638**	**(0.486, 0.837)**
Weak	**0.739**	**(0.657, 0.832)**		**0.742**	**(0.603, 0.913)**
**Physical health**					
Excellent	1.0		0.003		
Very good	1.004	(0.414, 2.434)			
Good	**0.365**	**(0.185, 0.719)**			
Okay	**0.785**	**(0.664, 0.928)**			
Weak	0.917	(0.818, 1.028)			
**BMI**					
Underweight	1.0		0.09	1.0	
Normal	1.142	(0.844, 1.546)		1.067	(0.743, 1.534)
Overweight	1.138	(0.833, 1.556)		0.860	(0.586, 1.263)
Obese	**1.391**	**(1.013, 1.909)**		1.158	(0.785, 1.707)
**Depression**					
Never	1.0		<0.001		
Some days	**0.641**	**(0.570, 0.722)**			
Few days	**0.653**	**(0.550, 0.775)**			
More than few days	**0.707**	**(0.578, 0.866)**			
**Anxiety**					
Never	1.0		<0.001		
Some days	**0.748**	**(0.661, 0.847)**			
Few days	**0.680**	**(0.579, 0.798)**			
More than few days	**0.654**	**(0.548, 0.780)**			
**Disability**					
Yes	1.0		0.41		
No	0.857	(0.593, 1.239)			
**Brushing**					
Never	1.0		<0.001	1.0	
Once a day	1.040	(0.831, 1.302)		1.099	(0.708, 1.705)
Twice a day	**0.793**	**(0.638, 0.987)**		1.563	(0.999, 2.447)
More than twice a day	0.827	(0.650, 1.054)		**1.984**	**(1.216, 3.239)**
**Dentist visit**					
Yes	1.0		0.93		
No	0.995	(0.893, 1.109)			
**Vigorous exercise**					
Never	1.0		<0.001		
Once a day	**1.191**	**(1.059, 1.340)**			
Twice a day	**1.298**	**(1.093, 1.541)**			
More than twice a day	**1.361**	**(1.112, 1.665)**			
**Moderate exercise**					
Never	1.0		<0.001		
Once a day	**1.605**	**(1.331, 1.934)**			
Twice a day	**1.363**	**(1.071, 1.734)**			
More than twice a day	**1.305**	**(1.118, 1.522)**			
**Light exercise**					
Never	1.0		<0.001	1.0	
Once a day	1.183	(0.985, 1.422)		1.345	(0.950, 1.906)
Twice a day	**1.381**	**(1.134, 1.683)**		**1.477**	**(1.023, 2.133)**
More than twice a day	**1.243**	**(1.088, 1.422)**		1.215	(0.958, 1.541)
**Smoking**					
Yes	1.0		<0.001	1.0	
No	**0.712**	**(0.621, 0.816)**		**1.454**	**(1.094, 1.932)**
**Shisha use**					
Yes	1.0		0.003		
No	**0.755**	**(0.626, 0.910)**			
**Cigar use**					
Yes	1.0		0.97		
No	0.990	(0.567, 1.727)			
**E-Cigarettes use**					
Yes	1.0		0.002		
No	**0.425**	**(0.245, 0.739)**			
**Chewing tobacco**					
Yes	1.0		0.09		
No	2.639	(0.868, 8.027)			
**Chronic diseases**					
Diabetes mellitus	**1.267**	**(1.018, 1.578)**	0.03		
hypertension	**1.397**	**(1.082, 1.803)**	0.01		
Hypercholesterolemia	1.094	(0.898, 1.333)	0.37		
CVD	**1.435**	**(1.000, 2.061)**	0.05		
Arthritis	**0.682**	**(0.525, 0.886)**	0.004		
Hepatitis	**1.566**	**(1.034, 2.373)**	0.03		
Cancer	1.009	(0.733, 1.388)	0.95	**2.053**	**(1.124, 3.751)**
Depression	0.788	(0.534, 1.164)	0.23		
Anxiety	0.824	(0.525, 1.294)	0.40		
Asthma	1.056	(0.829, 1.344)	0.66		

*Bold values indicate significant at the P < 0.05 level*.

The multivariable analysis identified age, gender, marital status, employment status, mental health status, dental hygiene, exercise level, smoking, and cancer as predictors of seatbelt use. Those who were 26–45 years old were 49% more likely to use a seatbelt than those who were 18–25 years old when adjusting for other covariates (Table 2). Women were 86% less likely to fasten their seatbelt, and married individuals were 38% more likely to use a seatbelt than those who were never married. Although employment status was positively associated with seatbelt use at the univariable level, the association was negative after adjusting for covariates.

While smokers were more likely to report seatbelt use, those who brush their teeth more than twice a day were 98% more likely to wear seatbelts. Respondents who reported weak mental health status were 26% less likely to wear seatbelts. Among chronic conditions, only cancer remained significant in the multivariable model, with patients diagnosed with cancer being twice as likely to report seatbelt use than those without a cancer diagnosis.

## Discussion

Our study found low seatbelt compliance among study participants despite initiatives being established to enforce compliance and increase awareness in the country. The low seatbelt use presented here is consistent with previous national literature (10, 13–16). Although most national studies have reported low seatbelt compliance, the exact estimates vary significantly from study to study. A study published in 2007 investigated the rate of seatbelt use in three Saudi cities, including Riyadh. The overall rate of seatbelt use was reported as 27.85% for drivers and 14.75% for front-seat passengers. Another study showed that 47% of drivers and 26% of front-seat passengers used seatbelts in the urban areas of Dammam (14). Both studies reported higher rates of seatbelt use among more experienced drivers who were older, married, more highly educated, and had higher income (14, 16).

Interventions implemented to improve traffic safety have been successful in reducing crashes and injuries in Saudi Arabia (19). However, the low compliance rate might be attributed to the recentness of introducing these preventive measures compared to other countries (20). Although a seatbelt law was established in 2000 (13), strict enforcement came years later in 2018, such as the installation of surveillance cameras to detect violations (12).

National studies show a higher compliance rate among older individuals (13, 21). Teenagers and young adults are less likely to fasten their seatbelts (16). This is similar to previous findings in other regional (17) and non-regional studies (22). Younger Qatari drivers and passengers were less likely to wear seatbelts than other age groups. Similarly, according to the 2011 Youth Risk Behavior Surveys in 38 states, only half of teenagers reported the consistent use of seatbelts (22). Similar findings have also been reported in Singapore (23). Therefore, interventions aimed at increasing compliance need to be designed and implemented while taking this group into account to improve traffic safety.

Use of seatbelts among females varies worldwide. In our study, women reported a lower rate of seatbelt use than male participants, which is similar to a finding reported over 6 years earlier (15). Singapore is one of the countries where women are less compliant than men in seatbelt use (23). On the other hand, females in the US report a higher rate of seatbelt use (89.6%) compared to males (81.9%) (22). This variation between countries might be attributed to socioeconomic factors or traffic education programs. In addition, many women ride in vehicles with personal drivers and thus occupy the rear seat. In KSA, there is no violation for not using a seatbelt while occupying the rear seat. Therefore, women may have responded from their experience as rear seat passengers and since it is not violation, they were more inclined to tell the truth. Further work is needed to understand the true compliance rate among Saudi women and to increase it for drivers as well as front and rear seats passengers. Furthermore, the changes in female seatbelt compliance since they started driving 2 years ago should also be examined (24).

In terms of marital status, married participants reported higher rates of seatbelt use than single participants. A similar finding was reported in a study conducted in the United Arab Emirates, which concluded that married individuals exhibited safer driving behavior and were more supportive of law enforcement policies (25). This might be attributed to factors like age or an inner sense of responsibility.

According to our findings, participants with mental illnesses reported low seatbelt compliance. Mental illnesses do not necessarily mean an inability to drive. However, functional impairment may be influenced by mental illnesses and can change an individual's driving behavior, which might endanger other road users (26). According to the literature, the presence of mental illness symptoms such as anxiety and depression might decrease road safety (27). Furthermore, a study done in Australia targeting young drivers identified depression as a predictor of risky behavior (28).

Participants with cancer were more compliant with seatbelt use. These patients may have adopted a healthier lifestyle that led to an improvement in their safety practices. Similar results were found in a study that examined risk behavior among teens with chronic conditions, such as cystic fibrosis and sickle cell disease. The study concluded that ill teenagers were more likely to fasten their seatbelts than their healthy counterparts (29). In the same manner, the higher compliance rate among survey participants who brush their teeth more than others may be explained by the possibility that they have adopted a healthier lifestyle. A 2017 study investigated factors that influence dental health behavior and found that adopting a healthy lifestyle was significantly associated with better oral hygiene (30).

Surprisingly, smokers were more likely to report seatbelt use. This finding is contrary to what has been reported previously (29). Moreover, a study conducted in Kuwait showed that smokers were less likely to buckle up and more likely to be involved in road traffic accidents (31). It is possible that social desirability bias has influenced smokers to be more likely to lie about seatbelt use than non-smokers. Leading to this observed result (32). Further studies are needed to confirm or disprove this finding.

Laws were recently implemented and led to increases in seatbelt usage due to the use of detection cameras (12). However, further enforcement is needed to reduce RTC, particularly because KSA is working to achieve the “2030 Vision,” in which one of the key performance indicators is reducing the mortality rate due to RTCs by 7% annually (31).

The study findings can support on-going efforts to improve traffic safety in KSA. Public health interventions could use these findings to target specific groups such as females and those with mental deficits. Such interventions include designing and implementing public health educational programs that focus on reaching a wider audience via avenues such as social media, which is very popular in KSA. In addition, our findings could serve as baseline for studies aiming to measure the impact of these public health interventions to ultimately improve traffic safety and public health.

This study has some limitations that need to be acknowledged in light of these findings. First, the study was based on a self-reported survey and thus has validity issues that are inherently associated with such modality. This may have resulted in overestimating the true prevalence of seatbelt compliance. However, self-reported estimates of several variables (e.g., smoking) are similar to those reported elsewhere (33). Furthermore, even if there is a self-reported bias, we expect it to be minimal because the seatbelt compliance reported here remains substantially lower than that in developed countries.

Second, the study population was limited to National Guard beneficiaries. Therefore, the generalizability of the findings should be limited to that population, which may differ from the general population. Third, we did not include a question to identify whether respondents answered based on being a driver or a passenger, which may influence the results. However, we have no reason to believe that a participant's answer may change since seatbelt use is mandated for both front-seat positions. Despite these limitations, the study is the first to use the SNB data, a major Saudi project that recruited a large number of participants. The population includes adults from different backgrounds, including military personnel, students, health care workers, and other community members, which are more likely to represent the underlying Saudi population.

In conclusion, seatbelt use remains low in the KSA and substantially lower than in developed countries. Teenagers, young adults, females, and those with mental disabilities were less likely to fasten their seatbelts. These findings could be used by public health programs to raise awareness about the need to increase seatbelt compliance and reduce traffic injuries. This study highlights the need for further investment in public health approaches focusing on injury prevention. Such interventions could include the design and implementation of public health educational programs that mainly focus on less compliant groups, such as females and those with mental health deficits. This could lead to increased seatbelt compliance and ultimately minimize road traffic injuries and its related health and economic consequences.

## Data Availability Statement

All datasets generated for this study are available from the institution upon reasonable request.

## Ethics Statement

The studies involving human participants were reviewed and approved by the King Abdullah International Medical Research Center Institutional Review Board. The patients/participants provided their written informed consent to participate in this study.

## Author Contributions

SA, TA, MA, and AAb drafted the initial manuscript, worked on the data acquisition, interpreted the data, and edited the manuscript. MB and AAlg assisted with data interpretation, reviewed, and revised the manuscript. AAlq developed the project, interpreted the data, supervised the work, and edited the manuscript. All authors read and approved the submitted version of the manuscript to be published.

## Conflict of Interest

The authors declare that the research was conducted in the absence of any commercial or financial relationships that could be construed as a potential conflict of interest.
